# A Novel Vasoactive Peptide “PG1” from Buffalo Ice-Cream Protects from Angiotensin-Evoked High Blood Pressure

**DOI:** 10.3390/antiox10030441

**Published:** 2021-03-12

**Authors:** Albino Carrizzo, Manuela Giovanna Basilicata, Giacomo Pepe, Kasper K. Sørensen, Michele Ciccarelli, Veronica Di Sarno, Antonio Damato, Eleonora Venturini, Anna Borrelli, Simona Musella, Mario Abate, Paola Di Pietro, Carmine Ostacolo, Pietro Campiglia, Carmine Vecchione

**Affiliations:** 1 Department of Medicine and Surgery, University of Salerno, 84081 Baronissi, SA, Italy; albino.carrizzo@gmail.com (A.C.); mciccarelli@unisa.it (M.C.); mabate@unisa.it (M.A.); pdipietro@unisa.it (P.D.P.); 2 IRCCS Neuromed, Vascular Pathophysiology Unit, 86077 Pozzilli, IS, Italy; antonio.damato85@gmail.com (A.D.); eleonora.venturini94@libero.it (E.V.); 3 Department of Pharmacy, University of Salerno, 84084 Fisciano, SA, Italy; mbasilicata@unisa.it (M.G.B.); gipepe@unisa.it (G.P.); vdisarno@unisa.it (V.D.S.); 4 Department of Chemistry, University of Copenhagen, 1870 Frederiksberg, Denmark; kso@chem.ku.dk; 5 Biomolecular Nanoscale Engineering Center, University of Copenhagen, 1870 Frederiksberg, Denmark; 6 University Hospital “San Giovanni di Dio e Ruggi D’Aragona”, via S. Leonardo, 1, 84131 Salerno, SA, Italy; direzione.sanitaria@sangiovannieruggi.it; 7 European Biomedical Research Institute of Salerno, 84131 Salerno, SA, Italy; s.musella@ebris.eu; 8 Department of Pharmacy, University of Naples Federico II, 80131 Napoli, NA, Italy; ostacolo@unina.it

**Keywords:** natural derived-peptide, cardiovascular, angiotensin II

## Abstract

Background: Arterial hypertension is the most important risk factor for cardiovascular diseases, myocardial infarction, heart failure, renal failure and peripheral vascular disease. In the last decade, milk-derived bioactive peptides have attracted attention for their beneficial cardiovascular properties. Methods: Here, we combined in vitro chemical assay such as LC-MS/MS analysis of buffalo ice cream, ex vivo vascular studies evaluating endothelial and smooth muscle responses using pressure myograph, and translational assay testing in vivo the vascular actions of PG1 administration in murine models. Results: We demonstrate that a novel buffalo ice-cream-derived pentapeptide “QKEPM”, namely PG1, is a stable peptide that can be obtained at higher concentration after gastro-intestinal digestions (GID) of buffalo ice-cream (BIC). It owns potent vascular effect in counteract the effects of angiotensin II-evoked vasoconstriction and high blood pressure levels. Its effects are mediated by the inhibitory effect on AT1 receptor leading to a downregulation of p-ERK½/Rac1-GTP and consequent reduction of oxidative stress. Conclusions: These results strongly candidate PG1, as a novel bioactive peptide for the prevention and management of hypertension, thus expanding the armamentarium of preventive strategies aimed at reducing the incidence and progression of hypertension and its related cardiovascular complications.

## 1. Introduction

Arterial hypertension (AH) is among the most critical risk factors for cardiovascular diseases and organ damage development [1]. Due to the dysregulated production of reactive oxygen species (ROS), oxidative stress plays a crucial pathophysiological role in the development of AH and its long-term complications, such as cardiovascular remodeling and atherosclerosis. In this regard, Angiotensin II (Ang II), the active peptide of the renin-angiotensin system (RAS), plays a key role in the development of AH by regulating several biological processes mainly through redox-sensitive signaling pathways [2,3]. In particular, in the vascular system, Ang II exerts a potent and acute vasoconstrictive effect and chronic cardiovascular remodelling with hypertrophy and fibrosis [4], mainly mediated by its interaction with Ang II type 1 receptor (AT1R) [3]. The acute and chronic activation of the AT1R involves the activation of NADPH oxidases (NOXs), multisubunit enzyme complexes with multiple and in part unknown regulatory interactions. Seven isoforms of NOX have been identified in eukaryotic cells so far, each distinguished by the identity of its catalytic subunit [5]. In particular, vascular tissues express NOX1, NOX2, and NOX5, known for their primary function of generating ROS and the hydrogen peroxide-generating enzyme NOX4. The activation of NOX1, NOX2 and NOX4 depend on association with a small GTPase Rac1, which is essential for the correct assembly of NADPH subunits. The regulation of these NOXs provides important positive and negative feedback regulatory mechanisms involved in the modulation of vascular system [6,7,8,9,10]. Owing to the critical role of oxidative stress in AH, the investigation on the potential effects of antihypertensive drugs on oxidative stress has been paid increasing attention by researchers. According to WHO data, AH is the leading cause of death globally, however, no more than 40% of the population has blood pressure in the normal range (DBP: 120–129 mmHg; DBP: 80–84 mmHg) [11]. The Angiotensin-converting enzyme (ACE) inhibitors and Angiotensin Receptor Blockers (ARBs) are the drugs of choice for the control of high blood pressure [12] and are indicated starting from grade 1 AH. Thus, categorically excluding those subjects with BP level at high normal range (SBP: 130–139 mmHg; DBP: 85–89 mmHg) and at increased risk for masked AH. This concept has made it necessary to identify new possible compounds that can be used to prevent and reduce the incidence of AH. Coined by Dr De Felice in 1989, the term “Nutraceutics” includes all those natural bioactive compounds with nutritional and pharmaceutical properties [13] that share the characteristic of health-promoting effects, such as polyphenols, carotenoids, polyunsaturated fatty acids, and natural derived-peptides. Compared to classical pharmacological therapy, natural bioactive compounds are not subject to specific government laws and can be used both as preventive agents and as adjuvants to traditional treatment. Several preclinical and clinical evidence demonstrated the most common commercial nutraceuticals’ high tolerability and safety profile [14]. Moreover, they improve the patient’s adherence, preferring natural-derived compounds to obtain long-term health benefits [15]. Thus, several studies have focused on identifying natural bioactive compounds with potential antihypertensive effects [16,17,18]. Milk and milk-derived products represent an important source of amino acids, proteins, fat, vitamins, calcium, fatty acids, and several other bioactive compounds needed for our cells’ biochemical and physiological functions [19]. During the last decade, numerous casein-derived peptides with cardiovascular properties have been identified and tested for their properties as antihypertensive compounds. However, most of these have failed during the translation in vivo, probably because they have been identified by protein hydrolysates of cheese sources, thus giving rise to a series of chemically unstable peptides for gastrointestinal digestion. To overcome this problem, we applied an in vitro simulated human gastrointestinal digestions (GID) using endogenous enzymes to mimic a buffalo ice-cream (BIC) matrix’s physiological process. Although many peptides have come-out through this experimental approach, we decided to investigate the peptide that emerged in more abundant quantities, assuming that in vivo would have been more likely to exert a biological action. In particular, we investigated the possible direct vascular effects of the new pentapeptide, namely PG1 (QKEPM), which originates from the GID of αS1-casein (f146-150). This work’s primary purpose was to investigate its possible vascular action both ex vivo, on the modulation of vascular tone, and in vivo testing its possible hemodynamic effects in a mice model of arterial hypertension induced by Ang II.

## 2. Materials and Methods

### 2.1. Characterization, Synthesis and Quantification of Buffalo Ice Cream (PG1) Peptide

The commercial buffalo ice cream product was kindly donated by the San Salvatore dairy factory (Capaccio, SA, Campania, Italy). The dairy product’s simulated gastrointestinal product was performed as previously described [20].

#### 2.1.1. Identification of PG1 Peptide in Buffalo Ice Cream Digest

LC-MS/MS analysis of buffalo ice cream digest were performed on a Shimadzu Nexera UHPLC system coupled online to an LCMS-IT-TOF mass spectrometer through an ESI source (Shimadzu, Kyoto, Japan). LC-MS analysis was carried out on Aeris^TM^ Peptide 100 × 2.1 mm × 1.7 μm column (Phenomenex, Bologna, Italy) thermostated at 60 °C. As mobile phase we have been used H_2_O and ACN both acidified by 0.1% (*v/v*) formic acid. The analysis was performed in gradient elution setting the flow rate at 0.5 mL min^−1^.

MS detection of bioactive peptides was operated in positive ionization mode and the full scan MS data were acquired in the range of 300–2000 *m/z*. The search of peptide sequences was performed using a SwissProt/UniProt database (database *Bubalus bubalis* release 2017).

#### 2.1.2. Fmoc-Based Solid-Phase Peptide Synthesis (SPPS) of PG1 Peptide

Synthesis of PG1 peptide (QKEPM) was performed according to the solid phase approach using standard Fmoc methodology, by Biotage^®^ Initiator + synthesizer.

The peptide was synthesized on a Fmoc-Met-TentaGel R-PHB resin (0.400 g, 0.21 mmol/g) previously deprotected with 20% piperidine/DMF (1 × 3 min, 1 × 10 min) at room temperature. The resin was then washed with DMF. The following protected amino acids: Fmoc-Pro-OH, Fmoc-Glu(OtBu)-OH, Fmoc-Lys(Boc)-OH and Fmoc-Gln(Trt)-OH were then added on to the resin stepwise. Each coupling reaction was accomplished using a 4-fold excess of amino acid with HBTU (3.6 eq) and HOAt (4 eq) in the presence of DIEA (7.2 eq). Amino acids were dissolved in DMF, and the coupling reactions were performed at 75 °C for 10 min. After each coupling step, the Fmoc protecting group was removed as described above. The N-terminal Fmoc group was removed, the resin was washed with DCM (5×), and the peptide was released from the resin with TFA/Anisole/H_2_O (90:5:5) plus tetraoctylammonium bromide (3% *p/v*) for 2 h in order to prevent the methionine oxidation. The peptide resin was precipitated with cold diethyl ether.

Finally, PG1 peptide was purified by Biotage^®^ Selekt Flash Purification System and analyzed by LCMS on Dionex Ultimate 3000 with Chromeleon 6.80SP3 software coupled to an ESI-MS (MSQ Plus Mass Spectrometer, Thermo, Waltham, MA, USA) (see supporting information Section S1 for description of chromatographic parameters). 

#### 2.1.3. Quantification of PG1 Peptide in Buffalo Ice Cream Digest

Quantitative analysis of PG1 peptide in GI digest of buffalo ice cream was performed on a Shimadzu Nexera UHPLC system coupled online with a LCMS-8050 (Shimadzu, Kyoto, Japan) equipped with an electrospray source (ESI). LC-MS data elaboration was performed by the Labsolutions^®^ software (Shimadzu). MS/MS analysis was conducted in selected reaction monitoring (SRM), employing the following transitions with 632.25 *m/z* as a precursor: 632.25–386.20 *m/z* (quantifying ion); 632.25–247.20 *m/z*; 632.25–222.15 *m/z* (see supporting information Section S2 for description of chromatographic parameters). 

### 2.2. Determination of Pharmacokinetic Properties of PG1 Peptide

#### 2.2.1. Chemical and Biochemical Stability of Pentapeptide

In order to determine pharmacokinetic properties of a synthetic peptide, we have evaluated its chemical stability during in vitro simulated GI digestion. In detail, we have recreated the enteric lumen by alkalinization at pH = 7.5 with 10 mM HCOONH_4_ buffer.

The peptide (5 µM) was incubated for 3 h and monitored by mass spectrometry experiments. On the other hand, the assessment of PG1 enzymatic stability was performed by treating the peptide with digestive enzymes. In detail, pentapeptide was incubated with pancreatin (from the porcine pancreas), chymotrypsin (from bovine pancreas) and bile salts. Digestive reactions were carried out at 37 °C for 180 min with 1:10 or 1:100 peptide/enzyme ratio, *w/w*. Finally, the mixture was centrifuged at 4000× *g* at 4 °C for 10 min, and PG1 peptide was collected at time 0 and at the end of the experiment and then analyzed by LC-MS/MS for the mass balance calculation.

#### 2.2.2. In Vitro Intestinal Transepithelial Transport Studies

##### Crystal Violet Assay for Determining Viability of Cultured Cells

To evaluate the effect of PG1 peptide on colorectal adenocarcinoma (Caco-2) cells, the cells were plated in 48-well plates at a density of 36 × 10^3^ cells/well. After 24 h, the Hanks’ Balanced Salt solution containing PG1 peptide at the indicated concentrations was added to the wells. The cells were examined after 3 h through microscope analysis. Finally, cells’ viability was assessed by the use of Crystal Violet Staining Solution.

All experiments were performed in triplicate, and the relative cell viability was expressed as a percentage in comparison with the untreated control cells.

##### Caco-2 Cell Monolayers Permeation Experiments

The colorectal adenocarcinoma (Caco-2) cell line was purchased from ATCC (Rockville, MD, USA). Cells were cultured in DMEM high glucose (4.5 g/L) with 2 mM L-glutamine and 10% (*v/v*) heat-inactivated fetal bovine serum. Cells were maintained at 37 °C in a humidified 5% CO_2_ atmosphere. Differentiation experiments were performed in a 12-well multiwell in transwell inserts (PET membrane, 0.4 µm pore size, 0.30 cm^2^ surface area), plating Caco-2 at 2.6 × 10^5^ cells/cm^2^ for 21 days in complete medium, changed every other day. By 21 days, cells become completely differentiated into enterocytic monolayers. 

The integrity of the monolayers was evaluated by means of the transepithelial electrical resistance (TEER) measurement using an EVOM2 epithelial voltohmmeter (World Precision Instruments, Sarasota, FL, USA). The apparent permeability coefficient (Papp) value of propranolol tested with the monolayer was 8.05 ± 0.16 × 10^−5^ cm/s, which was closely consistent with the value reported in the literature [21]. For the permeation assays, only intact monolayers with TEER higher than 300 Ω × cm^2^ were used. In brief, PET membranes were washed for 15–20 min at 37 °C with pre-warmed Hank’s balanced salt solution (HBSS) buffered with 25 mM HEPES and NaHCO_3_ (0.35 g/L) to adjust pH at 7.4, or with 10 mM methanesulfonic acid to adjust pH at 6.5. HBSS was added both to the apical (0.3 mL) and to the basolateral (0.9 mL) transwell compartments, as previously described [22]. 

For transport experiments, donor solution containing PG1 peptide was added to the apical compartment. Samples were collected from the receiving compartment at four different time points and from the donor compartment at time 0 and at the end of the experiment (180 min) for the mass balance calculation. Samples were stored at −20 °C until UHPLC-MS/MS analyses to measure the concentration of PG1 peptide in both compartments (see supporting information Section S3).

The apparent permeability coefficient (P_app_) was calculated according to the Equation (1). The donor concentration was recalculated by subtracting the cumulative amount transported to the receiver chamber for each time interval:

(1) $$C_{D}(t_{i})=C_{D}(t_{i}{-}_{1})-\frac{\left[C_{R}\left(t_{i}\right)-f\times C_{R}\left(t_{i-1}\right)\right]\times V_{R}}{V_{D}}$$

*C_D_* (*t_i_*) and *C_R_* (*t_i_*) (μM): donor and receiver chamber concentrations calculated at each sample occasion (*i*) from the donor and receiver concentrations at the previous occasion *C_D_* (*t_i_*_−1_) and *C_R_* (*t_i_*_−1_), respectively; *f* = 1 − *V_S_*/*V_R_*: sample replacement dilution factor; *V_S_* (cm^3^): sample volume; *V_R_* (cm^3^): receiver chamber volume; *V_D_* (cm^3^): donor chamber volume.

##### Immunofluorescence Analysis on Caco-2 Cell Monolayers

Integrity of monolayers was evaluated at the end of transport experiments fixing the PET membranes with 4% paraformaldehyde (PFA) for 15 min after a wash with PBS. Membranes were then incubated with blocking solution (0.1% Triton, 1% BSA, 0.02% sodium azide, 50 mM ammonium chloride) for 20 min at room temperature in the dark, followed by incubation with anti-zonulin 1 antibody (#402200, Invitrogen, Thermo Fisher Scientific, Waltham, MA, USA) at 2 μg mL^−1^ (RT, 2 h). Immunofluorescence was performed with Alexa Fluor 488 donkey anti-rabbit IgG (#A31573, Invitrogen) incubation (90 min, at 4 μg mL^−1^). Nuclei were counterstained with DAPI (1:2000). Cut membranes were mounted on slides with VectaMount solution (AQ Vector Laboratories, Burlingame, CA, USA). Slides were examined under a Nikon fluorescence inverted microscope (Nikon Instruments Europe, Firenze, Italy) and analyzed as described [23].

#### 2.2.3. Microsomal Stability Assay

2 μL of PG1 peptide (1 mM) were incubated with 183 µL of 100 mM phosphate buffer (pH 7.4), and 5 μL of 20 mg/mL human microsomes (Thermo Fisher Scientific, Bremen, Germany). After pre-incubation in a water bath for 5 min, the mixture was added with 10 μL of the 20 mM NADPH at further incubated at 37 °C for 60 min. The reaction was stopped with ice-cold methanol, centrifuged at 10,000 rpm at room temperature for 5 min and analyzed by LC-MS/MS. Three controls were used: T0 reference was obtained stopping the microsomal reaction by addition of the organic solvent immediately after incubation with microsomes. As positive and negative controls were used testosterone and PG1 solution incubated without NADPH. The extent of metabolism is expressed as a percentage of the parent compound turnover using the following equation:

(2) $$\%\;\mathrm{Parent}\;\mathrm{compound}\;\mathrm{turnover}\;=\;100\;-\;[\frac{\mathrm{concentration}\;\mathrm{at}\;60\;\min}{\mathrm{concentration}\;\mathrm{at}\;0\;\min}\;\times \;100]$$

#### 2.2.4. In Vivo Determination of PG1 Bioavailability

For the determination of serum concentration of pentapeptide in mice treated with PG1, blood samples were taken from the ventricle, performing a slow withdrawal to prevent the heart collapsing, to obtain a large quantity of high-quality blood. After collection, samples were rapidly centrifuged at 800 g to separate and collect serum. Extraction was conducted with ice-cold ACN plus 0.1% TFA (100 µL serum + 200 µL ACN). Samples were vortexed for 30 s and then centrifuged for 5 min at 10,000 rpm (Eppendorf Centrifuge model 5425). The supernatant was dried under nitrogen steam and reconstituted in 50:50 H_2_O/ACN. Calibration curve prepared by serial dilution of synthetic peptide solubilized in water in over the range 200–10 pM (y = 0.0167x + 0.7127, R^2^ = 0.9998, LOD = 2.8 pM, LOQ = 8.6 pM).

### 2.3. Animals

The data that support the findings of this study are available from the corresponding authors on reasonable request. Quantification of vasomotor response, blood pressure and molecular analyses were performed by a second individual who was blind to the animal’s genotype or the hypothesis that was being tested for each group. All experiments involving animals conformed to the Guide for the Care and Use of Laboratory Animals published by the US National Institutes of Health (NIH Publication No. 85-23, revised 2011). Male C57BL/6 mice (25 ± 0.7 g body weight) were generated in our animal facility. Male eNOS knockout mice (24 ± 1.0 g body weight) were obtained from Charles River Laboratories. All animals were randomly divided into control and treated groups. All efforts were made to minimize the number of animals used and their suffering. Mice (8 weeks old) were fed standard chow and water ad libitum. C57BL/6 mice were treated with angiotensin II in another experimental set, using osmotic minipumps (Alzet Model 2002; Alza Corp). For implantation of osmotic minipumps, mice were anaesthetized with 0.03 mL of a 2:1 mixture of ketamine (100 mg/mL i.m.; Aveco Co., Frankfurt, Germany) and xylazine (20 mg/mL i.m.; Miles Inc., Redwood City, CA, USA) and placed on an operating surface maintained at 38 °C. The interscapular region was shaved, and an osmotic minipump that contained angiotensin II (infusion rate 0.7 mg/Kg/day) inserted via a 1-cm incision to permit subcutaneous infusion. Sham-operated animals underwent an identical surgical procedure but with an empty osmotic pump implanted. No animals died during treatments.

### 2.4. Vascular Reactivity Studies

Second-order branches of the mesenteric arterial tree were removed from animals to perform vascular studies. Vessels were placed in a pressure myograph system filled with Krebs solution at pH 7.4 at 37 °C as previously described [24]. Some mesenteric arteries mounted on a myograph system were pre-treated for 1h with PG1 at 1, 10, 50, 100 mM before data for dose–response curves were obtained. Quantification of vasomotor response was performed by a second individual who was blind to the animal’s genotype and/or the hypothesis that was being tested for each group. In all vascular experiments, precontraction was obtained by administering increasing doses of angiotensin II (10^−^^9^ to 10^−^^6^ M) to get a similar pre-contraction level [24]. 

### 2.5. Assesment of Oxidative Stress 

Lucigenin assay was used to measure oxidative stress in mouse mesenteric arteries in control (untreated), angiotensin II (Ang II; 10^−^^6^ mol/L) or Ang II plus the different concentration of PG1 preincubated for 1 h. The chemiluminescence that occurred over the next 5 min in response to the addition of 100 mmol/L NADPH was recorded (Tecan Infinite M200 Pro). In preliminary experiments, homogenates alone, without NADPH’s addition, gave only minimal signals, and NADPH did not evoke lucigenin chemiluminescence in the absence of homogenate. 

Dihydroethidium (DHE) staining was used to graphically display the levels of oxidative stress in mice mesenteric arteries as previously described [25]. Briefly, vessels were stained with 5 mmol/L DHE for 20 min and observed under a fluorescence microscope (Zeiss, Oberkochen, Germany). Images were acquired by a digital camera system. 

### 2.6. BP Measurements in Mice

Blood pressure was evaluated by noninvasively measured in conscious mice with a tail-cuff method, using a BP-2000 instrument (Visitech Systems) as previously described [24]. Briefly, animals were placed in a holder on a temperature-controlled platform (kept at 37 °C), and recordings were performed in steady-state conditions. BP values were averaged from at least 3 consecutive measurements. 

### 2.7. Protein Extraction and Immunoblot Analysis

For protein extraction, pooled mesenteric arteries were lysed in a buffer containing 150 mmol/L NaCl, 50 mmol/L Tris-HCl (pH 8.5), 2 mmol/L EDTA, 1% *v/v* NP-40, 0.5% *w/v* deoxycholate, 10 mmol/L NaF, 10 mM sodium pyrophosphate, 2 mmol/L PMSF, 2 g/mL leupeptin, and 2 g/mL aprotinin, pH 7.4. Lysates were incubated on ice for 15 min and then centrifuged at 38,000× *g* for 30 min at 4 °C to collect the supernatant. Protein concentration was measured using a dye-binding protein assay kit (Bio-Rad) and reading to the spectrophotometer at a wavelength of 595 nm. Immunoblotting was performed as previously described [25], using the following antibodies: anti-bactin (Abcam, ab49900; mouse monoclonal, 1:4000), anti-phospho-ERK1/2 (Santa Cruz, sc-136521; mouse monoclonal 1:800), anti-AT1 receptor (Santa Cruz, sc-57036; mouse monoclonal 1:1000), anti-Rac1-GTPγ (STA-401-1, Cells Biolab Inc., 1:800). Secondary antibodies (1:3000) were purchased from Amersham Life Sciences (GE Healthcare). Bands were visualized with enhanced chemiluminescence (ECL, Amersham Life Sciences), according to the manufacturer’s instructions. Immunoblotting data were analysed using ImageJ software (developed by Wayne Rasband, NIH, Bethesda, MD, USA) to determine the density of the bands. 

### 2.8. Statistical Analysis

All data are given as mean ± SEM. 2-way ANOVA followed Bonferroni post hoc test for multiple comparisons was used to evaluate the effects on endothelium-dependent vasorelaxation in response to acetylcholine. *t*-test and 1-way ANOVA followed by Bonferroni post hoc test were used to compare 2-independent groups or more than 2-independent groups, respectively. 

A *p*-value of less than 0.05 was considered statistically significant, and was reported in figures as followed *, *p* < 0.05; **, *p* < 0.001; ***, *p* < 0.0001 or ^#^, *p* < 0.05; ^##^, *p* < 0.001; ^###^, *p* < 0.0001.

Prism statistical software was used to perform statistical analysis (Graphpad, La Jolla, CA, USA).

## 3. Results

### 3.1. Identification and Synthesis of PG1 Peptide

Oxidative stress is considered the leading cause of endothelial dysfunction, mostly through reducing nitric oxide bioavailability. Therefore, we evaluated the potential antioxidant effect of buffalo ice-cream digest against oxidative stress induced by Ang II in mice mesenteric arteries. For this purpose, a dihydroethidium (DHE) probe, able to react with superoxide and hydrogen peroxide, was used to detect oxidative stress levels. 

To reveal the main antioxidant molecule in the buffalo ice-cream digest, we evaluated the peak’s primary structure possessing the highest area percent in the UV chromatogram of the digesta. An abundant peptide (5.05 ± 0.07 µg mL^−1^; 7.59 ± 0.11 µM) was revealed and its primary structure was tentatively identified as pentapeptide deriving from the hydrolysis of αS1-casein (f146-150, Gln-Lys-Glu-Pro-Met, QKEPM, PG1 peptide, Table 1).

PG1 peptide was synthesized according to the solid phase approach to obtain the amounts necessary for its pharmacological evaluation. We realized that the critical issue was represented by the scarce stability of methionine residue, quickly undergoing oxidation, in the reaction conditions used during the synthesis. Met oxidation is a known side reaction in peptide synthesis, especially during the removal of protecting groups and peptide cleavage from the resin under acidic conditions. The thioether moiety on the methionine side chain can be oxidized by acid catalysis to corresponding Methionine sulfoxide (MetO) or even sulfone (MetO_2_). To prevent the oxidation of the Methionine residues and improve the final yields (Figure S1) during the trifluoroacetic acid (TFA) cleavage step in the SPPS, we have employed a cleavage cocktail containing tetraoctylammonium bromide, an appropriate reducing agent with good solubility in TFA and anisole as scavengers for bromine (Br_2_) [26]. 

### 3.2. Evaluation of the Vascular Effects of PG1 on Mice Resistance Arteries 

**Figure 1:**
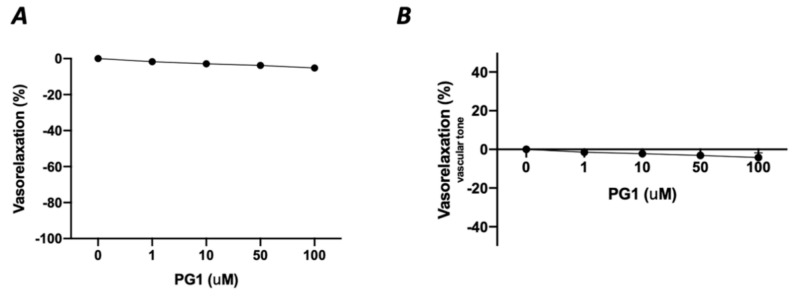
(**A**) Assessment of possible vascular effect of PG1 at increasing doses 1, 10, 50 and 100 μM on mice mesenteric arteries preconstricted with phenylephrine (10^−9^ to 10^−5^ M). (**B**) Evaluation of possible direct vascular action of increasing doses of PG1 (1, 10, 50 and 100 μM) on uncontracted equilibrated mice mesenteric arteries.

**Figure 2:**
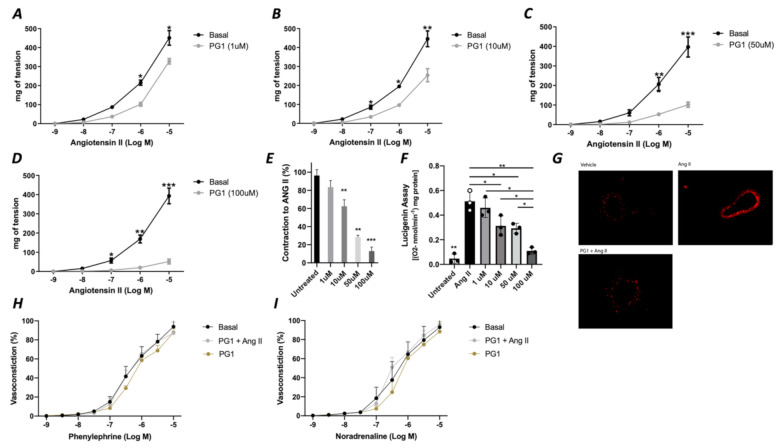
(**A**–**D**) Effects of PG1 at different doses 1 μM (**A**), 10 μM (**B**), 50 (**C**) and 100 μM (**D**) on angiotensin II-evoked vasoconstriction in mice mesenteric arteries compared to vehicle treated alone. (**E**) Percentage of reduction of vasoconstriction at different dosage of PG1 (1, 10, 50 and 100 μM). (**F**) Oxidative stress measured by lucigenin assay on vessels treated with Angiotensin II alone, or ore-incubated with PG1 for 1 h at different doses 1, 10, 50 and 100 μM before Angiotensin II stimulation (10^−5^). (**G**) Representative micrographs of Dihydroetidium staining in mesenteric arteries treated with vehicle, with angiotensin-II (10^−5^) for 30 min of with PG1 (100 μM) plus Angiotensin II. H-I) Vasoconstrictive responses to increasing doses of (**H**) phenylephrine or (**I**) noradrenaline, respectively, in basal condition (without stimuli), after PG1 (100 μM) pretreatment, and after pretreatment with PG1 (100 μM) plus Ang II (10^−7^ M). *, *p* < 0.05; **, *p* < 0.001; ***, *p* < 0.0001.

In the first phase of the study, we tested the potential direct effect of PG1 on the vascular tone’s modulation. The administration of increasing doses of PG1 (1, 10, 50 and 100 mM) on phenylephrine-constricted mesenteric arteries did not evoke any direct vascular action (Figure 1A). The same effect was observed in pressure myograph-normalized uncontracted vessels, in which vascular tone was not modified by PG1 exposure (Figure 1B). Subsequently, we performed a series of experiments to assess its potential role in modulating angiotensin II-evoked vascular dysfunction. One hour of pre-incubation with PG1 resulted in the containment of vasoconstrictive stimuli induced by Ang II (Figure 2A–D). Of note, the comparison of the reduction of vasoconstriction percentages showed complete inhibition of mesenteric arteries contraction at 100 mM of PG1 (Figure 2E).

### 3.3. Assessment of PG1 Antioxidant Effect 

Considering that the main vascular effect of Ang II is mediated by the release of the high amount of ROS, we assessed the role of PG1 on the modulation of oxidative stress. Interestingly, measurement of oxidative stress by lucigenin assay revealed that PG1 pretreatment significantly reduced ROS production in a dose-dependent manner (Figure 2F). The statistical analysis revealed that at 100 mM of PG1, there was almost a complete abolition of ROS production. This result was also confirmed by DHE staining, which showed that at the highest concentration of PG1, mice mesenteric arteries were wholly protected from Ang II-induced ROS generation (Figure 2G). Of note, PG1 treatment does not affect phenylephrine or norepinephrine-evoked vasoconstriction (Figure 2H,I), thus confirming the peptide’s specificity to interact with the molecular mechanism involved in angiotensin signaling.

### 3.4. Pharmacokinetics Properties of PG1 Peptide

#### 3.4.1. Pancreatic Digestion 

The oral delivery of therapeutic peptides is a challenge in pharmaceutical science, with proteolysis as a significant barrier. Proteolysis is the primary elimination pathway for the vast majority of peptides. In particular, peptides are susceptible to proteases or peptidases due to amide bonds in their structures. GI tract contains many endo- and exopeptidases, enzymes that cleave the peptide bonds and act synergistically to degrade proteins and peptides. Both luminally secreted enzymes (e.g., pepsins, trypsin, and chymotrypsin) and brush border membrane-bound enzymes (e.g., endopeptidases, aminopeptidases, and carboxypeptidase) play essential roles in peptide proteolysis [27]. Indeed, the greatest threat to therapeutic peptides lies in the small intestine lumen, which contains gram quantities of peptidases secreted from the pancreas and cellular peptidases from the mucosal cells, which are sloughed continuously off from the villi [28]. Based on these considerations, we have evaluated the chemical and enzymatic stability of PG1 peptide, simulating the typical conditions of intestinal digestion. Initially, we verified its chemical stability by incubating PG1 at 37 °C in 10 mM HCOONH_4_ solution at Ph = 7.5. Mass spectrometry studies showed high peptide stability for up to 3 h. Enzymatic stability was assessed by incubation with pancreatin and chymotrypsin in two different peptide/enzyme (1:10 or 1:100 *w/w*). LC-MS/MS again analysed the intestinal digests. Our results showed that PG1 peptide is not hydrolysed in the experimental conditions used.

#### 3.4.2. Intestinal Bioavailability

To assess PG1 peptide bioavailability, its transmembrane permeability was evaluated through Caco2 cell monolayers. We used Caco-2 cells for in vitro study because, when grown on permeable filters, are recognized as the golden standard for in vitro prediction of intestinal drug permeability and absorption. Indeed, when cells are in culture, slowly differentiates into monolayers with a differentiated phenotype with many functions of the small intestinal villus epithelium.

The amount of PG1 peptide permeated through the monolayer increased approximately linearly, in time-dependent (0–180 min) manner demonstrating good permeability (Papp = 0.20 – 5.36 ± 0.94 × 10^−6^ cm/s). 

The intestinal absorption of PG1 peptide takes place through a passive diffusion mechanism, in consideration of the efflux ratio (Papp,_b/a_/Papp,_a/b_) that is less than 1. Moreover, the transmembrane uptake of PG1 peptide is independent of the proton gradient. In order to evaluate the effects of PG1 peptide on Caco-2 monolayers integrity and cell vitality, we performed immunofluorescence analysis on transwell inserts at the end of transport studies.

Caco-2 cell monolayers integrity was preserved upon PG1 peptide treatment at the concentration tested as confirmed by tight junction protein zonulin-1 expression (green) and cell vitality (Figure S2).

#### 3.4.3. Microsomal Stability

First pass metabolism is an essential factor that affects the oral bioavailability of drugs. Drugs absorbed from the gastrointestinal tract are first delivered to the liver by the portal vein. A fraction of the drug is then metabolized in the liver before it even reaches the systemic circulation. The liver is the major metabolizing organ for endogenous substrates as well as exogenous drugs. Several in vitro tools are available to study drug candidates’ metabolic fate, including isolated fresh or cryopreserved hepatocytes, liver slices, and subcellular fractions such as S9 fractions liver and microsomes. In particular, the liver’s microsomes are an enriched source of cytochrome P450 (CYP) and flavin monooxygenases (FMO) enzymes. Therefore, we have evaluated the microsomal stability of PG1 (1 mM) incubated with 20 mg/mL human microsomes and 20 mM NADPH at 37 °C for 60 min. After stopping the reaction by adding ice-cold methanol, the supernatants were collected and analyzed by LC-MS/MS. The extent of metabolism was expressed as a percentage of the parent compound turnover, compared to the control obtained by the addition of organic solvent immediately after incubation with microsomes. Our data showed that the parent PG1 peptide turnover percentage was about 13.18 ± 3.6%, indicating good metabolic stability. 

### 3.5. Evaluation of the Haemodynamic Effect of PG1 during In Vivo Treatment 

**Figure 3:**
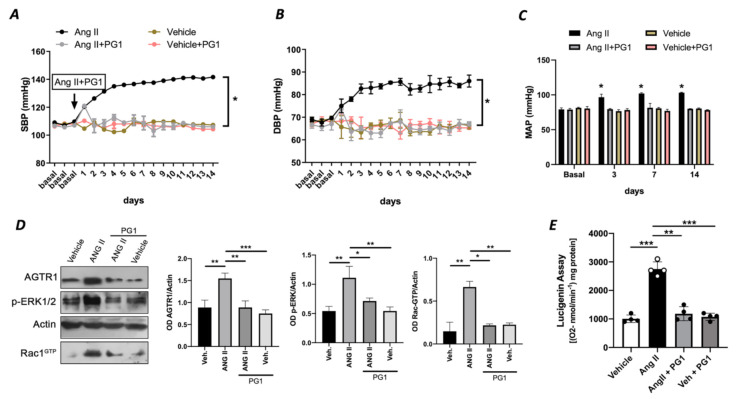
(**A**) Systolic blood pressure (SBP) and (**B**) diastolic blood pressure (DBP) and (**C**) mean arterial pressure (MAP) measured by tail-cuff method in wild-type mice daily treated with i.p injection of vehicle or PG1 (10 mg/Kg), or in wild-type angiotensin (Ang) II-infused mice daily treated with gavage administration of vehicle or PG1 (10 mg/Kg) mice. Values are means ± SEM. (**D**) Representative immunoblots conducted on extracts of mesenteric arteries excised at the end of in vivo (14th day) treatment from wild-type mice daily treated with gavage administration of vehicle or PG1 (10 mg/Kg), or in wild-type angiotensin (Ang) II-infused mice daily treated with gavage administration of vehicle or PG1 (10 mg/Kg) mice. (**E**) Oxidative stress measured by lucigenin assay in mice mesenteric arteries obtained at the end of in vivo treatment. *, *p* < 0.05; **, *p* < 0.001; ***, *p* < 0.0001.

To rule out an exclusive in vitro effect of PG1 on Ang II signalling, we evaluated in vivo the potential impact of PG1 in mice treated with chronic infusion of Ang II. PG1 administration normalized both systolic and diastolic blood pressure in response to Ang II treatment (Figure 3A,B). The effect was maintained for all the interval of PG1 daily administration, thus strengthening the peptide’s selective role on Ang II-mediated vascular signalling. Accordingly, the measurement of mean arterial pressure (MAP), which represents the average of blood pressure in a single cardiac cycle, clearly showed the beneficial action of PG1 in Ang II-treated mice compared to vehicle-treated. Moreover, the same treatment performed did not evoke any haemodynamic modification in normotensive mice (Figure 3A–C). 

At the molecular level, PG1 downregulated the expression of Ang II type I receptor only in Angiotensin II-treated mice (Figure 3D) but not in the normotensive counterpart. Additionally, we observed an upregulation of phospho-ERK½ and Rac1-GTP in mesenteric arteries isolated from Ang II-treated mice but restored to near basal levels when treated with PG1 (Figure 3D). Interestingly, the analysis of oxidative stress in mesenteric arteries collected at the end of treatment showed a significant reduction of ROS in Ang II plus PG1-treated mice compared to Ang II- alone treated ones (Figure 3E). Finally, the serum concentration of pentapeptide in mice treated with PG-1 was about 17.0 ± 2.49 pM as determined by LC-MS/MS. 

### 3.6. PG1 Effects on Vascular Function of Resistance Vessels 

**Figure 4:**
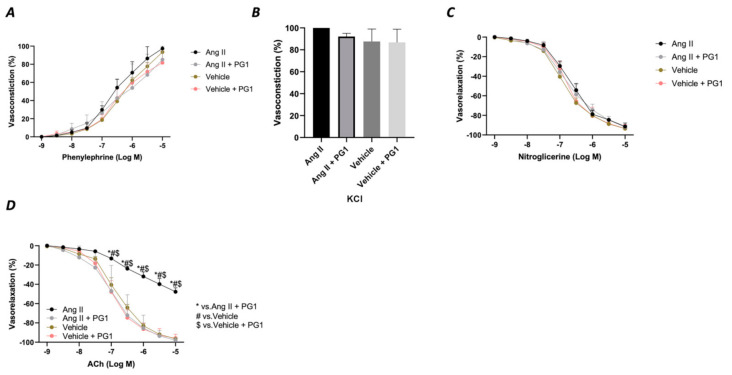
Assessment of vascular response of mice mesenteric arteries obtained at the end of in vivo (14th day) treatment from wild-type mice daily treated with gavage of vehicle or PG1 (10 mg/Kg), or in wild-type angiotensin (Ang) II-infused mice daily treated by gavage administration of vehicle or PG1 (10 mg/Kg) mice to (**A**) phenylephrine, (**B**) Potassium chloride, (**C**) Nitroglycerine (**D**) acetylcholine.

Considering the massive effect of PG1 on Ang II-evoked high blood pressure, we evaluated the vascular response of mesenteric arteries at the end of in vivo chronic treatment with the peptide. The analysis of the vasoconstrictive response to phenylephrine and potassium chloride revealed no modification on the contractile function of the vessels (Figure 4A,B). A similar effect was observed during the vasorelaxant response to nitroglycerine, which directly evokes a smooth muscle-dependent vasorelaxation (Figure 4C). In contrast, the analysis of endothelial vasorelaxation prompted by acetylcholine revealed a marked endothelial dysfunction in vessels obtained from Ang II-treated mice compared to vehicle-treated. Notably, PG1 administration protected against endothelial dysfunction evoked by chronic Ang II treatment (Figure 4D).

### 3.7. Evaluation of PG1 Vascular and Hemodynamic Effects in eNOS Deficiency Condition 

**Figure 5:**
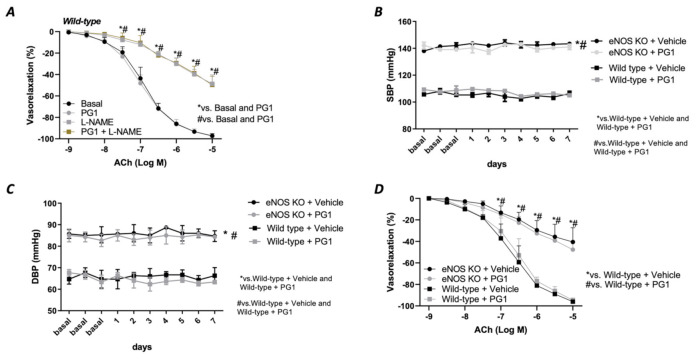
(**A**) Evaluation of ex vivo vascular reactivity of PG1 (100 μM for 1 h) in wild-type mice treated with eNOS inhibitor, L-NAME (300 μM for 30 min). (**B**,**C**) Tail-cuff measurement of systolic and diastolic blood pressure in wild-type and eNOS knock-out mice gavage-treated with vehicle or PG1 (10 mg/Kg) for 7 days. (**D**) Evaluation of endothelial-dependent relaxation in mesenteric arteries obtained from wild-type and eNOS knockout mice excised at the end of in vivo treatment (7th day) with vehicle or PG1.

Prompted by the strong hemodynamic effect evoked by PG1 under the condition of elevated Ang II circulating levels, we decided to investigate its impact on the eNOS deficiency condition. To pursue this aim, we tested its in vitro action in the presence of a selective eNOS inhibitor, L-NAME. As reported in Figure 5, pretreatment with PG1 during eNOS inhibition did not exert any beneficial effect on the altered Ach-mediated vasorelaxation (Figure 5A), leading us to suppose a complete exclusion of nitric oxide in the PG1 vascular action. To definitively confirm these results, we treated eNOS- deficient mice model with PG1 for 1 week. Blood pressure monitoring revealed a complete inefficacy to reduce both systolic and diastolic pressure in this model (Figure 5B,C). Moreover, the endothelial-dependent vasorelaxation assessment revealed a similar dysfunction between vehicle- and PG1-treated mice (Figure 5D), clearly demonstrating the lack of nitric oxide involvement in PG1 signalling.

## 4. Discussion

This study demonstrated that a novel pentameric peptide, namely PG1, isolated from in vitro simulated gastric and intestinal digestion (SGID) of buffalo ice-cream, owns potent vascular effect in counteracting the effects of angiotensin II-evoked vasoconstriction. In particular, its effects are mediated by the inhibitory effect on the AT1 receptor leading to a downregulation of p-ERK½/Rac1-GTP and the consequent reduction of oxidative stress in experimental models. Milk is a good source of many critical bioactive proteins and peptides. Dietary intake of dairy proteins is related to decrease the risk of cardiovascular diseases [19]. In the last decade, several milk-derived bioactive peptides have been identified and proposed as useful compounds against chronic diet-related diseases, particularly non-communicable diseases such as obesity, cardiovascular disease and diabetes. In particular, the most extensively studied mechanism underlying the antihypertensive effects of milk is represented by ACE-inhibition [29,30,31]. Several casein-derived peptides, such as VPP, IPP, FFVAP, KVLPVP, YKVPQL, TTM- PLW, AVPYPQR, and FFVAPFPEVFGK, have shown significant ACE inhibitory activity [32,33]. In particular, VPP and IPP peptides have already been exploited in commercial products, demonstrating antihypertensive effects in humans/patients [34,35]. Other bioactive peptides with ACE2 inhibitory activity have been identified in the fermentation product of cheese, sour milk or yoghurt showing a higher hypotensive effect in spontaneously hypertensive rats (SHR) [36,37]. Unfortunately, all these bioactive peptides have been identified in protein hydrolysates from various cheese sources, allowing to obtain peptides that are chemically unstable to gastrointestinal absorption and unable to exert an action in vivo. Differently from this approach, and following our previous study in which we have identified a novel bioactive pentapeptide isolated from buffalo ricotta cheese [38], we applied in vitro simulated human GID [39], using endogenous enzymes to mimic a physiological process on buffalo ice-cream matrix. This technique owns enormous advantages since it leads to the generation of resistant to digestion products after oral administration and allows identifying more-potent bioactive products [16]. We identified and characterized the most abundant peptide’s vascular effect through this experimental approach, namely PG1 (αS1-casein, f146-150, QKEPM) obtained from the GID of buffalo ice-cream, which sequence, so far, has never been reported. The choice to use the mouse mesenteric arteries, in the first part of the study is based on the evidence that the mesenteric artery represents the prototype of the resistance arteries involved in the regulation of arterial pressure unlike windkessel such as aorta. Here, we demonstrate that PG1 was able to inhibit Ang II-evoked vasoconstriction in mice mesenteric arteries completely. This effect was mediated by a potent inhibitory effect of PG1 on oxidative stress production. Before in vivo analysis, we have evaluated the pharmacokinetics properties of PG1 peptide in different in vitro models. Our results highlighted the peptide’s high stability to the chemical and enzymatic conditions of the intestinal lumen and incubation with liver microsomes, possibly due to the presence in its primary structure of a proline residue. This aminoacid introduces a conformation constraint reducing the peptide’s flexibility and, consequently, susceptibility to proteolysis [40]. Finally, the bioavailability of PG1 peptide was evaluated in intestinal transport studies through Caco-2 cell monolayer. Our results suggested a moderate intestinal absorption of peptide mainly through a passive diffusion mechanism. To assess its possible in vivo efficacy, we employed the Ang II-induced model of hypertension, which mimics the pathological characteristics of human hypertension, including vascular dysfunction [16]. As expected, while the chronic release of Ang II induced an increase in both systolic and diastolic blood pressure, daily oral administration of PG1 completely restored normal blood pressure levels. The inhibitory effect versus Ang II persists during all the administration period, suggesting a direct interference with the Ang II vascular signalling. It is well-known that AT1 receptors are responsible for the hypertensive effects of Ang II, inducing vasoconstriction and producing a high amount of free oxygen radicals by activating the NADPH oxidase enzyme. Our molecular analysis revealed that prolonged treatment with PG1 during high circulating levels of Ang II completely blocked AT1R overexpression, phospho-ERK1/2 levels and the consequent reduction of Rac-1 GDP/GTP exchange, which represents a prerogative mechanism for the activation of different types of NADPH oxidase enzyme [5], such as NOX1, NOX2 and NOX4, in vascular tissue [41]. Several studies suggested that the prolonged vasoconstriction induced by high circulating levels of Ang II, often reflects the alteration of vascular physiological response due to oxidative stress [42,43]. Through its AT1 receptor, Ang II favor a dysregulation of redox state promoting the inactivation of nitric oxide (NO) [44], the most crucial molecule contributing to vascular homeostasis [45]. Herein, the effect of PG1 reflects its capability to counteract the Ang II-evoked oxidative stress specifically. Indeed, PG1 does not interfere with α1, and α2 receptors mediated vasoconstriction, thus strengthening its specific role on the downregulation of AT1 receptor. In the last years, the area of functional foods and dietary supplements has attracted much attention from researchers focused on discovering novel bioactive peptides due to their recognition as useful tools for improving health and preventing chronic diseases [46]. Here, the use of a workflow based on the extraction of peptides after simulated GID, led us to identify a novel pentameric peptide, PG1, encrypted in the parent protein sequence (a-casein), which is delivered by digestion, absorbed by intestinal cells, and transported into the blood where it accomplishes their biological activity in counteracting the AT1 receptor stimulation by Ang II. It is well known that although a potentially reversible cause of hypertension can be identified in less than 10% of patients, the overall high prevalence of hypertension means that secondary forms can affect millions of patients worldwide [47]. In this regard, a dysregulation of ACE enzyme and overexpression of AT1R have been reported in pre-hypertensive patients [48]. In 2003, “pre-hypertension” has been defined as blood pressure ranging from 120–139 mmHg systolic and 80-89 mmHg diastolic [49], highlighting the concept that “pre-hypertension” does not represent a disease category. Thus, lifestyle modifications are the only practices prescribed for this category to reduce hypertension risk. Unfortunately, many pre-hypertensive individuals turn into full-blown hypertensive individuals within two years of their first diagnosis [50]. This has led the foundation to activate the first trial of preventing hypertension “TROPHY” [51]. This study demonstrated that transient pre-hypertensive treatment (TPT) with angiotensin II type 1 receptor (AT1R) blocker compared with placebo successfully delayed new onset of hypertension in a cohort of pre-hypertensive subjects within 2 years. However, TROPHY study was contested because it was considered a promoter of the early use of antihypertensive drugs [52], thus leaving unresolved the protective question about the antihypertensive pre-treatment adults aimed at reducing the overt onset of hypertension.

## 5. Conclusions

Using chemical and biological in vitro and in vivo approaches, we demonstrated that a novel buffalo ice-cream-derived pentapeptide “QKEPM”, namely PG1, obtained at higher concentration after gastro-intestinal digestions (GID) of buffalo ice-cream (BIC), owns potent vascular protective activity that recapitulates the cardiovascular action of ARBs. Its capability to counteract the Ang II-evoked vascular dysfunction and high blood pressure inhibiting overexpression and activation of AT1R/p-ERK1/2/Rac1-GTP signalling, strongly candidate PG1, as a novel potential peptide to be used as a preventive strategy aimed at preventing the incidence of overt hypertension and its related vascular complications. 

## 6. Study Limitation and Perspectives

Although the data here obtained demonstrate the beneficial effects of PG1 on vascular function and blood pressure regulation in a mice model of AH, further studies are needed to dissect the molecular mechanism involved and aim to identify the specific NADPH oxidase isoform involved. Certainly, after a complete evaluation of its bioactivity, safety, tissue distribution and pharmacokinetic analyses, we will seek to translate PG1 into the clinical scenario, mainly in all those in a pre-hypertension state, whose therapeutic tools are currently limited. 

Our mirage is to give life to a new natural-derived peptide, which may help prevent the onset of arterial hypertension and related cardiovascular complications.

## Figures and Tables

**Table 1:** Main peptides released during GI digestion of ice cream sample.

Protein	RT (min)	Mass	Error ppm	Amino Acid	Identified Peptide	Length
α_S1_-casein	3.265	631.2999	0.6	146–150	Q.QKEPM.I	5
α_S1_-casein	8.382	551.2227	−1.9	172–176	L.DAYPS.G	5
α_S1_-casein	9.825	674.2759	−3.0	99–104	K.EDVPSE.R	6
β-lactoglobulin	11.268	564.2544	−1.6	168–172	L.SFNPT.Q	5
β-casein	12.125	574.2751	1.0	172–176	M.FPPQS.V	5
β-casein	12.312	634.2962	−1.4	208–212	L.YQEPV.L	5
α_S1_-casein	12.498	570.3013	−0.9	125–129	L.EIVPN.L	5
ĸ-casein	12.925	559.2853	−0.9	138–142	K.TEIPT.I	5
β-casein	15.215	603.2904	−0.3	129–133	K.YPVEP.F	5
β-lactoglobulin	15.892	627.3228	−4.0	65–70	L.KPTPEG.D	6
β-casein	18.335	575.3431	0.4	149–153	L.HLPLP.L	5
β-casein	19.678	851.4905	−1.2	76–83	Y.PFPGPIPK.S	8
α_S1_-casein	22.055	754.3861	−0.3	195–201	F.SDIPNPI.G	7
β-casein	24.028	750.3588	−0.4	129–134	K.YPVEPF.T	6
β-casein	24.735	529.2900	0.0	218–222	R.GPFPI.I	5
β-casein	25.515	1000.5229	1.0	208–216	L.YQEPVLGPV.R	9
β-casein	26.125	1113.6222	0.5	74–83	L.VYPFPGPIPK.S	10
β-casein	30.342	1226.7063	−0.2	73–83	S.LVYPFPGPIPK.S	11
α_S1_-casein	33.015	904.4694	−0.8	39–46	F.FVAPFPEV.F	8
β-casein	36.248	977.5546	−1.5	84–92	K.SLPQNIPPL.T	9

## Data Availability

The data presented in this study are available on request from the corresponding author. The data are not publicly available due to the development of patent application on PG1 peptide.

## Supplementary Materials

The following are available online at https://www.mdpi.com/2076-3921/10/3/441/s1, Section S1: Purification and Characterization of Synthetic PG1 Peptide, Section S2: Quantification of PG1 Peptide in GI Digest of Buffalo Ice Cream, Section S3: Determination of PG1 Peptide in Apical and Basolateral Compartments of Caco-2 Cell Monolayers. Figure S1: Synthetic scheme (a), chromatographic profile (b), MS/MS fragmentation pattern (c) and UV spectrum (d) of PG1 peptide, Figure S2: (A) Fluorescence micrograph of the Caco-2 cell monolayers. Caco-2 cell monolayers treated with PG1 peptide (25 μM, 3h) from TEER experiments were stained for tight junction protein expression of zonulin-1 (FITC, green). Nuclei were counterstained with DAPI (blue). Pictures are representative of two independent experiments. Original magnification 200X. (B) Caco-2 cells were examined after 3 h of PG1 peptide treatment (25 μM) through microscope analysis. Light microscope images are representative of three independent experiments. Original magnification 200X. (C) Caco-2 cells were cultured for 3 h with PG1 (25 μM), before Crystal violet assay. Results are expressed as mean (± SD), and are representative of three independent experiments.

## References

[1] Weycker D., Nichols G.A., O’Keeffe-Rosetti M., Edelsberg J., Khan Z.M., Kaura S., Oster G. (2007). Risk-Factor Clustering and Cardiovascular Disease Risk in Hypertensive Patients. Am. J. Hypertens..

[2] Touyz R.M. (2004). Reactive oxygen species, vascular oxidative stress, and redox signaling in hypertension: What is the clinical significance?. Hypertension.

[3] Touyz R.M. (2005). Intracellular mechanisms involved in vascular remodelling of resistance arteries in hypertension: Role of angiotensin II. Exp Physiol..

[4] Harvey A., Montezano A.C., Lopes R.A., Rios F., Touyz R.M. (2016). Vascular Fibrosis in Aging and Hypertension: Molecular Mechanisms and Clinical Implications. Can. J. Cardiol..

[5] Carrizzo A., Forte M., Lembo M., Formisano L., Puca A.A., Vecchione C. (2014). Rac-1 as a new therapeutic target in cerebro- and cardio-vascular diseases. Curr. Drug Targets.

[6] Drummond G.R., Sobey C.G. (2014). Endothelial NADPH oxidases: Which NOX to target in vascular disease?. Trends Endocrinol. Metab..

[7] Sedeek M., Nasrallah R., Touyz R.M., Hebert R.L. (2013). NADPH oxidases, reactive oxygen species, and the kidney: Friend and foe. J. Am. Soc. Nephrol..

[8] Burtenshaw D., Hakimjavadi R., Redmond E.M., Cahill P.A. (2017). Nox, Reactive Oxygen Species and Regulation of Vascular Cell Fate. Antioxidants.

[9] Zafari A.M., Ushio-Fukai M., Akers M., Yin Q., Shah A., Harrison D.G., Taylor W.R., Griendling K.K. (1998). Role of NADH/NADPH oxidase-derived H2O2 in angiotensin II-induced vascular hypertrophy. Hypertension.

[10] Vecchione C., Patrucco E., Marino G., Barberis L., Poulet R., Aretini A., Maffei A., Gentile M.T., Storto M., Azzolino O. (2005). Protection from angiotensin II-mediated vasculotoxic and hypertensive response in mice lacking PI3Kgamma. J. Exp. Med..

[11] Williams B., Mancia G., Spiering W., Rosei E.A., Azizi M., Burnier M., Clement D.L., Coca A., de Simone G., Dominiczak A. (2018). 2018 ESC/ESH Guidelines for the management of arterial hypertension. Eur. Heart J..

[12] Ives C.W., Oparil S. (2019). What is the first choice for blood pressure treatment?. Lancet.

[13] Carrizzo A., Izzo C., Forte M., Sommella E., Di Pietro P., Venturini E., Ciccarelli M., Galasso G., Rubattu S., Campiglia P. (2020). A Novel Promising Frontier for Human Health: The Beneficial Effects of Nutraceuticals in Cardiovascular Diseases. Int. J. Mol. Sci..

[14] Cicero A.F.G., Colletti A. (2018). An update on the safety of nutraceuticals and effects on lipid parameters. Expert Opin. Drug Saf..

[15] Dima C., Assadpour E., Dima S., Jafari S.M. (2020). Bioavailability of nutraceuticals: Role of the food matrix, processing conditions, the gastrointestinal tract, and nanodelivery systems. Compr. Rev. Food Sci. Food Saf..

[16] Carrizzo A., Conte G.M., Sommella E., Damato A., Ambrosio M., Sala M., Scala M.C., Aquino R.P., De Lucia M., Madonna M. (2019). Novel Potent Decameric Peptide of Spirulina platensis Reduces Blood Pressure Levels Through a PI3K/AKT/eNOS-Dependent Mechanism. Hypertension.

[17] Drevet P. (2017). Evidence of antihypertensive effect of a land snail (Helix aspersa) by-product hydrolysate-Identification of involved peptides. Ann. Cardiol. Angeiol..

[18] Dabarera M.C., Athiththan L.V., Perera R.P. (2015). Antihypertensive peptides from curd. Int. Q. J. Res..

[19] McGregor R.A., Poppitt S.D. (2013). Milk protein for improved metabolic health: A review of the evidence. Nutr. Metab..

[20] Pepe G., Pagano F., Adesso S., Sommella E., Ostacolo C., Manfra M., Chieppa M., Sala M., Russo M., Marzocco S. (2017). Bioavailable Citrus sinensis extract: Polyphenolic composition and biological activity. Molecules.

[21] Chan C.O., Jing J., Xiao W., Tan Z.X., Lv Q.Y., Yang J.Y., Chen S.B. (2017). Enhanced Intestinal Permeability of Bufalin by a Novel Bufalin-Peptide-Dendrimer Inclusion through Caco-2 Cell Monolayer. Molecules.

[22] Hubatsch I., Ragnarsson E.G.E., Artursson P. (2007). Determination of drug permeability and prediction of drug absorption in Caco-2 monolayers. Nat. Protoc..

[23] Ranieri R., Ciaglia E., Amodio G., Picardi P., Proto M.C., Gazzerro P., Laezza C., Remondelli P., Bifulco M., Pisanti S. (2018). N6-isopentenyladenosine dual targeting of AMPK and Rab7 prenylation inhibits melanoma growth through the impairment of autophagic flux. Cell Death Differ..

[24] Carrizzo A., Moltedo O., Damato A., Martinello K., Di Pietro P., Oliveti M., Acernese F., Giugliano G., Izzo R., Sommella E. (2020). New Nutraceutical Combination Reduces Blood Pressure and Improves Exercise Capacity in Hypertensive Patients Via a Nitric Oxide-Dependent Mechanism. J. Am. Heart. Assoc..

[25] Persico M., Masarone M., Damato A., Ambrosio M., Federico A., Rosato V., Bucci T., Carrizzo A., Vecchione C. (2017). Non alcoholic fatty liver disease and eNOS dysfunction in humans. BMC Gastroenterol..

[26] Huang H., Rabenstein D.L. (1999). A cleavage cocktail for methionine-containing peptides. J. Pept. Res..

[27] Di L. (2015). Strategic approaches to optimizing peptide ADME properties. Aaps. J..

[28] Woodley J.F. (1994). Enzymatic barriers for GI peptide and protein delivery. Crit. Rev. Drug Carr. Syst..

[29] Abdel-Hamid M., Otte J., De Gobba C., Osman A., Hamad E. (2017). Angiotensin I-converting enzyme inhibitory activity and antioxidant capacity of bioactive peptides derived from enzymatic hydrolysis of buffalo milk proteins. Int. Dairy J..

[30] Shazly A.B., He Z.Y., El-Aziz M.A., Zeng M.M., Zhang S., Qin F., Chen J. (2017). Fractionation and identification of novel antioxidant peptides from buffalo and bovine casein hydrolysates. Food Chem..

[31] Shazly A.B., Mu H.B., Liu Z.M., Aziz M.A., Zeng M.M., Qin F., Zhang S., He Z.Y., Chen J. (2019). Release of antioxidant peptides from buffalo and bovine caseins: Influence of proteases on antioxidant capacities. Food Chem..

[32] Nakamura Y., Yamamoto N., Sakai K., Okubo A., Yamazaki S., Takano T. (1995). Purification and Characterization of Angiotensin I-Converting Enzyme-Inhibitors from Sour Milk. J. Dairy Sci..

[33] Ariyoshi Y. (1993). Angiotensin-Converting Enzyme-Inhibitors Derived from Food Proteins. Trends Food Sci. Tech..

[34] Donkor O.N., Henriksson A., Singh T.K., Vasiljevic T., Shah N.P. (2007). ACE-inhibitory activity of probiotic yoghurt. Int. Dairy J..

[35] Papadimitriou C.G., Vafopoulou-Mastrojiannaki A., Silva S.V., Gomes A.M., Malcata F.X., Alichanidis E. (2007). Identification of peptides in traditional and probiotic sheep milk yoghurt with angiotensin I-converting enzyme (ACE)-inhibitory activity. Food Chem..

[36] Tonouchi H., Suzuki M., Uchida M., Oda M. (2008). Antihypertensive effect of an angiotensin converting enzyme inhibitory peptide from enzyme modified cheese. J. Dairy Res..

[37] Ai L.Z., Guo B.H., Zhang H., Wu Z.J., Chen W., Wang Y.Y., Tang J.A. (2008). Isolation and antihypertensive effect of exopolysaccharides from Lactobacillus casei LC2W. Milchwissenschaft.

[38] Pepe G., Basilicata M.G., Carrizzo A., Adesso S., Ostacolo C., Sala M., Sommella E., Ruocco M., Cascioferro S., Ambrosio M. (2019). beta-Lactoglobulin Heptapeptide Reduces Oxidative Stress in Intestinal Epithelial Cells and Angiotensin II-Induced Vasoconstriction on Mouse Mesenteric Arteries by Induction of Nuclear Factor Erythroid 2-Related Factor 2 (Nrf2) Translocation. Oxid. Med. Cell Longev..

[39] Basilicata M.G., Pepe G., Sommella E., Ostacolo C., Manfra M., Sosto G., Pagano G., Novellino E., Campiglia P. (2018). Peptidome profiles and bioactivity elucidation of buffalo-milk dairy products after gastrointestinal digestion. Food Res. Int..

[40] Quiros A., Contreras M.D., Ramos M., Amigo L., Recio I. (2009). Stability to gastrointestinal enzymes and structure-activity relationship of beta-casein-peptides with antihypertensive properties. Peptides.

[41] Carrizzo A., Vecchione C., Damato A., di Nonno F., Ambrosio M., Pompeo F., Cappello E., Capocci L., Peruzzi M., Valenti V. (2017). Rac1 Pharmacological Inhibition Rescues Human Endothelial Dysfunction. J. Am. Heart Assoc..

[42] Gaspari T.A., Vinh A., Jones E.S., Widdop R.E. (2012). Ganging up on Angiotensin II Type 1 Receptors in Vascular Remodeling. Hypertension.

[43] Xu J.A., Carretero O.A., Liao T.D., Peng H.M., Shesely E.G., Xu J.X., Liu T.S., Yang J.J., Reudelhuber T.L., Yang X.P. (2010). Local angiotensin II aggravates cardiac remodeling in hypertension. Am. J. Physiol. Heart.

[44] Zhou M.S., Adam A., Raij L. (2001). Interaction among angiotensin II, nitric oxide and oxidative stress. J. Renin Angiotensin Aldosterone Syst..

[45] Forte M., Conti V., Damato A., Ambrosio M., Puca A.A., Sciarretta S., Frati G., Vecchione C., Carrizzo A. (2016). Targeting Nitric Oxide with Natural Derived Compounds as a Therapeutic Strategy in Vascular Diseases. Oxid Med. Cell Longev..

[46] Chakrabarti S., Guha S., Majumder K. (2018). Food-Derived Bioactive Peptides in Human Health: Challenges and Opportunities. Nutrients.

[47] Carey R.M., Muntner P., Bosworth H.B., Whelton P.K. (2018). Prevention and Control of Hypertension JACC Health Promotion Serie. J. Am. Coll Cardiol.

[48] Baumann M., Sollinger D., Roos M., Lutz J., Heemann U. (2010). Prehypertensive Preconditioning Improves Adult Antihypertensive and Cardioprotective Treatment. J. Pharm. Exp..

[49] McNiece K.L., Poffenbarger T.S., Turner J.L., Franco K.D., Sorof J.M., Portman R.J. (2007). Prevalence of hypertension and pre-hypertension among adolescents. J. Pediatr..

[50] Julius S., Nesbitt S., Egan B., Kaciroti N., Schork M.A., Grozinski M., Michelson E., Grp T.S. (2004). Trial of Preventing Hypertension-Design and 2-year progress report. Hypertension.

[51] Kaplan N.M. (2004). Trial of Preventing Hypertension (TROPHY)-Design and 2-year progress report. Hypertension.

[52] Meltzer J.I. (2006). A specialist in clinical hypertension critiques the trophy trial. Am. J. Hypertens..

