# Supplementary material for: A Novel Vasoactive Peptide “PG1” from Buffalo Ice-Cream Protects from Angiotensin-Evoked High Blood Pressure
Source: Antioxidants (Basel). 2021 Mar 12;10(3):441. doi: 10.3390/antiox10030441 (PMC8002072; doi:10.3390/antiox10030441)
Supplement: Supplementary file 1 [file antioxidants-10-00441-s001.pdf]

Supplementary information

Expanded Materials and Methods section

Section S1. *Purification and characterization of synthetic PG1 peptide*

The separation was carried out on Biotage® Sfär Bio C18 Duo (25 g, 300 Å, 20 µm), with detection at 215 and 280 nm. The flow rate was set to 35 mL min^{-1}, and the injection volume was 5 mL (2 mg mL^{-1}). The mobile phase consisted of (A) H_2O and (B) ACN both acidified by TFA 0.1% (v/v). Analysis was performed in gradient elution as follows: 2 CV, isocratic to 5% B; 4 CV, 5-35%B; 1 CV, 35-100%; 2 CV, isocratic to 100% B; then 3 CV for column re-equilibration.

The peptide analyzed on C4 column (150 × 4.6 mm × 5 µm, 300 Å) with a flow rate of 1.0 mL min^{-1}. The following solvent system was used: solvent A, water and solvent B, acetonitrile both containing 0.1% formic acid. The column temperature was set at 42 °C, and the peptide was eluted using a linear gradient from 5%-100% of solvent B in 9 min.

Section S2. *Quantification of PG1 peptide in GI digest of buffalo ice cream*

The separation was carried on a on BIOshell™ A160 Peptide ES C18 100 × 2.1 mm × 2.7 µm (Supelco, Bellefonte PA, USA) employing as mobile phase A) H_2O and B) ACN both acidified by formic acid 0.1% v/v, with the following gradient: 0-2.0 min, isocratic to 2% B; 2-5.0 min, 2-70% B; 5-5.01 min, 70-95%; 5.01-7.0 min, isocratic to 95% B; then eight minutes for column re-equilibration. The flow rate was set to 0.5 mL min^{-1}. Column oven was set to 30 °C, and 10 µL of samples were injected. Dwell time: 20 ms, Interface temperature: 250 °C, Desolvation line temperature: 200 °C, Heat Block temperature: 300 °C, Heating gas flow: 10 L/min, Nebulizing gas flow: 3.0 L/min. Calibration curve was obtained solubilizing in water the synthetic peptide in a concentration range of 12.5-0.5 µg mL^{-1}. Linear regression was used to generate calibration curve (y= 0.000004x + 0.121306, LOD= 0.16 µg mL^{-1}, LOQ= 0.48 µg mL^{-1}, R^2 = 0.9998).

Section S3. *Determination of PG1 peptide in apical and basolateral compartments of Caco-2 cell monolayers*

For the quantification of PG1 peptide in donor and receiver chamber, was employed as external standard the synthetic peptide. The stock solution was prepared in fresh HBSS, the calibration curve was obtained in a concentration range of 1000-8 nM with eight concentration levels, and triplicate injection of each level was run. Peak areas of PG1 peptide were plotted against corresponding concentrations. Linear regression was used to generate calibration curve (y = 0.00894x – 4.49519, LOD= 16.7 nM, LOQ= 50.6 nM) with R^2 values was ≥ 0.9991. MS/MS analysis of propranolol was conducted in SRM, employing the following transitions with 260.10 m/z as a precursor: 260.10-116.10 m/z (quantifying ion); 260.10-183.15 m/z (qualifying ion). For its quantification, the calibration curve was obtained in a concentration range of 250-7 nM (y = 0.00015x + 10.15063, with R^2 values ≥ 0.998, LOD= 12.0 nM, LOQ= 36.3 nM).

Figure S1

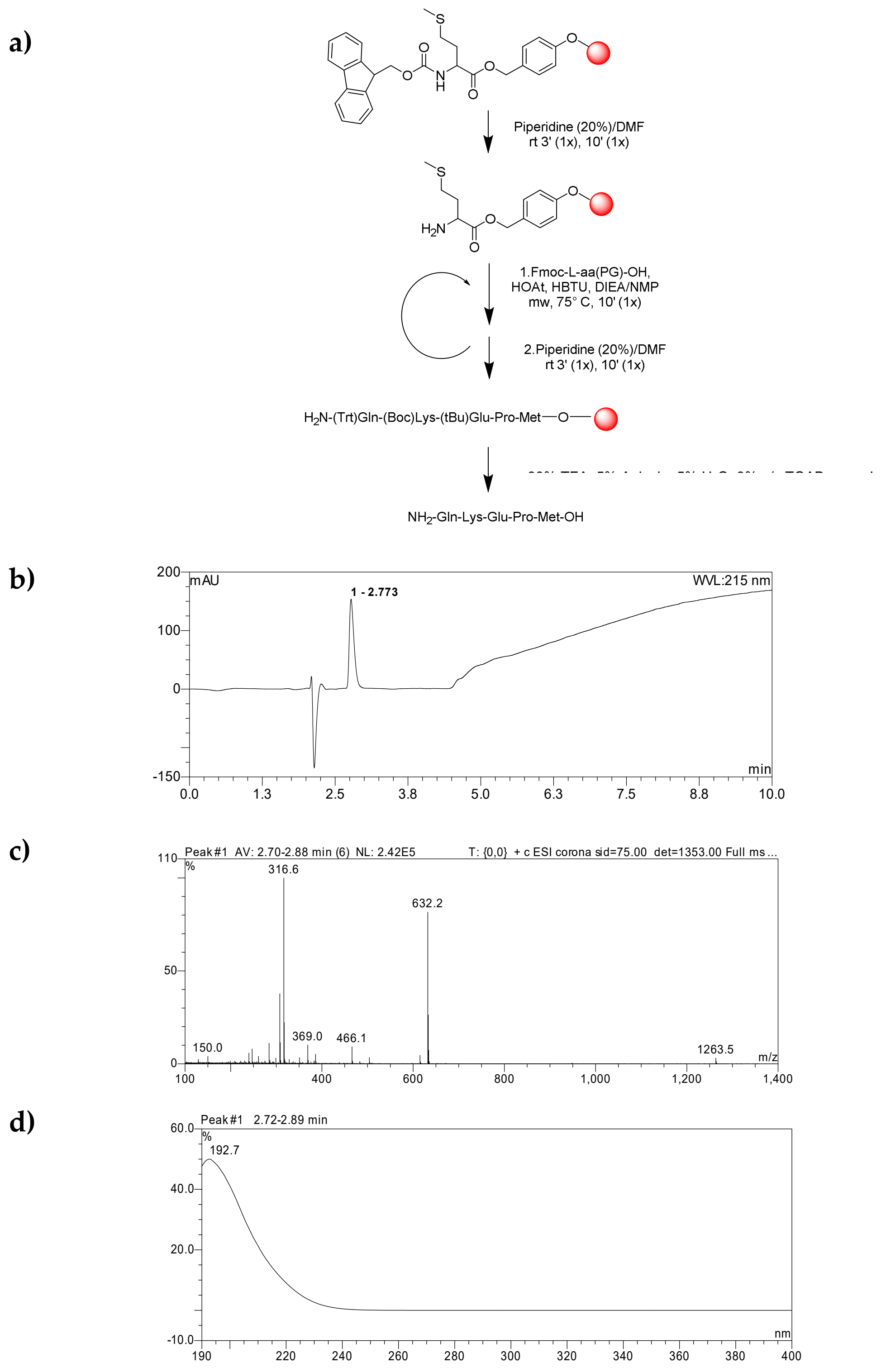

Figure S1. Synthetic scheme (**a**), chromatographic profile (**b**), MS/MS fragmentation pattern (**c**) and UV spectrum (**d**) of PG1 peptide.

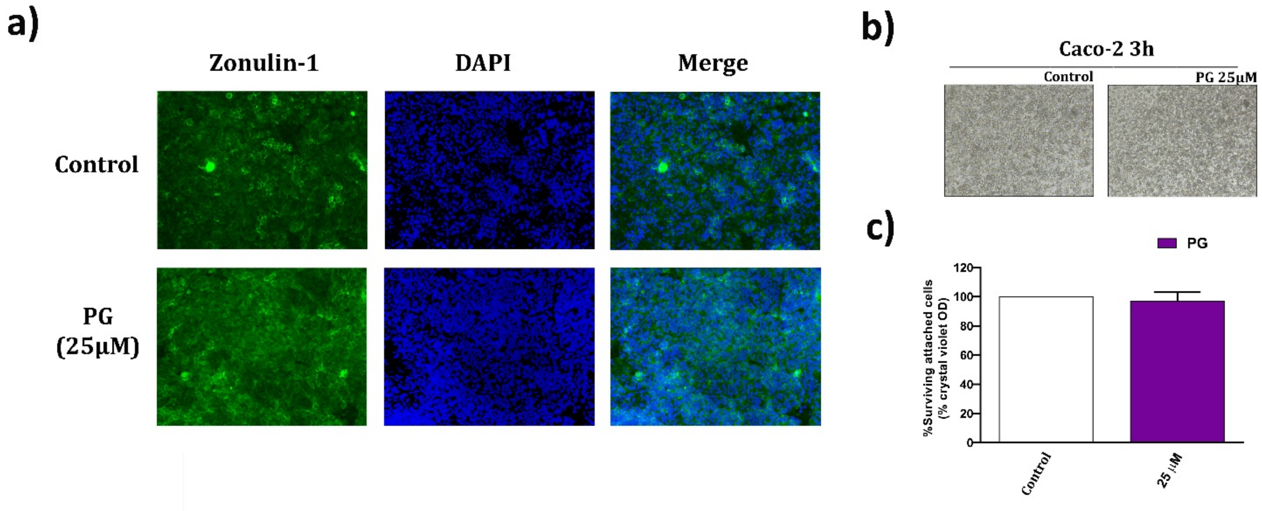

Figure S2. (A) Fluorescence micrograph of the Caco-2 cell monolayers. Caco-2 cell monolayers treated with PG1 peptide (25 µM, 3h) from TEER experiments were stained for tight junction protein expression of zonulin-1 (FITC, green). Nuclei were counterstained with DAPI (blue). Pictures are representative of two independent experiments. Original magnification 200X. **(B)** Caco-2 cells were examined after 3 hours of PG1 peptide treatment (25 µM) through microscope analysis. Light microscope images are representative of three independent experiments. Original magnification 200X. **(C)** Caco-2 cells were cultured for 3 hours with PG1 (25 µM), before Crystal violet assay. Results are expressed as mean (± SD), and are representative of three independent experiments.
